# Population genetics structure of glyphosate-resistant Johnsongrass (*Sorghum halepense* L. Pers) does not support a single origin of the resistance

**DOI:** 10.1002/ece3.671

**Published:** 2013-08-24

**Authors:** Luis Fernández, Luis Alejandro de Haro, Ana J Distefano, Maria Carolina Martínez, Verónica Lía, Juan C Papa, Ignacio Olea, Daniela Tosto, Horacio Esteban Hopp

**Affiliations:** 1 Instituto de Biotecnología, Instituto Nacional de Tecnología Agropecuaria (INTA Castelar)N. Repetto y Los Reseros, 1686, Hurlingham, Argentina; 2 Facultad de Ciencias Exactas y Naturales, Universidad de Buenos Aires (UBA)1424, Ciudad Universitaria, Argentina; 3 Estación Experimental Agropecuaria Oliveros, Instituto Nacional de Tecnología AgropecuariaRutan Km 353, CP: 2206, Oliveros, Santa Fe, Argentina; 4 Sección Malezas, Estación Experimental Agroindustrial Obispo ColombresAV. Williams Cross, 3150, Las Talitas, Tucumán, Argentina

**Keywords:** EPSPS, glyphosate, herbicide resistance, microsatellites, *Sorghum halepense*, weed epidemiology

## Abstract

Single sequence repeats (SSR) developed *for Sorghum bicolor* were used to characterize the genetic distance of 46 different *Sorghum halepense* (Johnsongrass) accessions from Argentina some of which have evolved toward glyphosate resistance. Since Johnsongrass is an allotetraploid and only one subgenome is homologous to cultivated sorghum, some SSR loci amplified up to two alleles while others (presumably more conserved loci) amplified up to four alleles. Twelve SSR providing information of 24 loci representative of Johnsongrass genome were selected for genetic distance characterization. All of them were highly polymorphic, which was evidenced by the number of different alleles found in the samples studied, in some of them up to 20. UPGMA and Mantel analysis showed that Johnsongrass glyphosate-resistant accessions that belong to different geographic regions do not share similar genetic backgrounds. In contrast, they show closer similarity to their neighboring susceptible counterparts. Discriminant Analysis of Principal Components using the clusters identified by K-means support the lack of a clear pattern of association among samples and resistance status or province of origin. Consequently, these results do not support a single genetic origin of glyphosate resistance. Nucleotide sequencing of the 5-enolpyruvylshikimate-3-phosphate synthase (EPSPS) encoding gene from glyphosate-resistant and susceptible accessions collected from different geographic origins showed that none presented expected mutations in aminoacid positions 101 and 106 which are diagnostic of target-site resistance mechanism.

*Sorghum halepense* (L.) Pers (Johnsongrass) is considered one of the most troublesome weeds in the world (Holm et al. 1977). Its control proved to be rather difficult because of its reproductive biology. It is rather established that its main strategy of reproduction in agricultural ecosystems is either by clonal dispersion of rhizome fragments through cultural practices (ploughing and tilling) or by delivery of seeds after self-pollination (Warwick and Black 1983). This reproduction strategy would theoretically lead, in the long run, to a decrease in genetic diversity and an increased degree of homozygosis. Moreover, it has been reported that only 5% of cross-fertilization occurs even in fields where plants are closely spaced (Tarr 1962; Warwick and Black 1983). However, no DNA markers were available to confirm these data at those times, nor have they been used to corroborate these observations yet. Thus, genomic studies applied to population genetics in this species are still rather few.

In spite of its economic importance and broad and valuable ecological knowledge regarding invasion strategies, little is known about its population genetics structure and dynamics, probably owing to the difficulties of dealing with polyploid genetics. There is consensus that *S. halepense* originated from hybridization between *S. propinquum* and *S. bicolor* ssp. *arundinaceum* followed by chromosome doubling (De Wet 1978). Accordingly, *S. halepense* is a tetraploid containing one subgenome in common with *S. bicolor,* for this reason, plenty of the molecular markers developed were able to be used for Johnsongrass.

Moreover, the recent annotation of cultivated *S. bicolor* genome sequence (Paterson et al. 2009) provides a resource of DNA markers for *S. halepense*. Paterson et al. (1995) have already used RFLP markers to locate 3 QTL related to weediness. Much more recently, these same molecular markers have been used to study the potential risk associated with the use of genetically modified sorghum through gene escape after hybridization between *S. bicolor* and *S. halepense* (Morrell et al. 2005). Chang et al. (2007) have used microsatellites (single sequence repeats, SSR) as well as cytogenetics to study the genetic relationship with *S. bicolor*. Wu and Huang (2006) have also applied SSR to develop a synteny map with *S. bicolor* to help marker-assisted introgression of traits into cultivated sorghum. Guo et al. (2008) have worked with them to study the genetic diversity in quarantined *S. halepense*. Finally, Jessup et al. (2012) have compared different polyploid *Sorghum* species, including *S. halepense*, employing SSR markers. However, none of these studies have carried out a population genetics analysis of Johnsongrass in relationship with herbicide-resistance propagation and epidemiology using molecular markers.

Studies of the genetic structure of weed populations may turn useful for their control, owing that genetic diversity affects the sustainability of control methods (Sterling et al. 2004). It has been reported that weeds with a high degree of asexual reproduction are often better controlled by biological agents than sexually reproducing weeds (Burdon and Marshall 1981), which is an interesting alternative for weeds that have developed resistance to common chemical herbicides as in the case of Johnsongrass. Furthermore, high levels of diversity could compromise not only the efficiency of control by biological agents (Okoli et al. 1997) but also of chemical herbicides (Costa and Appleby 1976) or mechanical means (Glaze 1987). Some ecologists hypothesized that the ability of Johnsongrass seeds to partially survive digestion by birds would be a way to ensure dispersion through long distances helping to colonize new environments (Holm et al. 1977). In addition to bird vectors, agricultural practices leading to contamination of agricultural crop seeds with Johnsongrass seeds seem to be another powerful way to ensure dispersal and mixture of genetic lineages. These factors would promote a rapid dispersion and establishment of a strong adaptive trait such as herbicide resistance, supporting a single origin hypothesis of resistance genes. This work tests this line of thinking.

Since 1974, glyphosate has been increasingly used in various weed control schemes and the increased adoption of genetically engineered glyphosate-resistant crops has accelerated its widespread use becoming the most widely used herbicide in world agriculture (Duke and Powles 2008; Powles 2008). One of the main reasons for glyphosate adoption was its effective control of recalcitrant weeds, which places Johnsongrass at the top of weed control with this herbicide. As a consequence of this intense selection, glyphosate resistance has evolved in several weed species including Johnsongrass (Owen 2008; Powles 2008; Vila-Aiub et al. 2008; Powles and Yu 2010; Riar et al. 2011). The mechanistic basis of resistance showed that the most expected mechanisms (but not necessarily the most frequently observed for Johsongrass) are target-site-based resistance because of mutations of the 5-enolpyruvylshikimate-3-phosphate synthase (EPSPS) coding sequence or upregulation of its expression level (Powles and Yu 2010). Hence, EPSPS*-*encoding sequence mutations, including serine, threonine, or alanine substitutions at Pro-106 (Pro-106-Ser/Thr/Ala), a highly conserved region of the EPSPS gene, have been reported in glyphosate-resistant *Eleusine indica* and *Lolium* spp. (Baerson et al. 2002; Pérez-Jones et al. 2007; Vila-Aiub et al. 2008; Preston et al. 2009; Powles and Yu 2010; Kaundun et al. 2011; González-Torralva et al. 2012). Also, a Gly-101-Ala substitution in the EPSPS-encoding sequence confers glyphosate resistance in various plant crops (Padgette et al. 1991; Pline-Srnic 2006; Funke et al. 2009; Powles and Yu 2010). In addition to EPSPS gene point mutations, EPSPS gene amplification in glyphosate-resistant *Amaranthus palmeri* and *Lolium perenne* (Gaines et al. 2010; Salas et al. 2012) and basal increase in EPSPS mRNA in *Conyza canadensis* have been documented (Dinelli et al. 2006). On the other hand, nontarget-site mechanisms have been also reported. Actually, this is the case for the glyphosate-resistant Johnsongrass evolved in Salta (northwestern Argentina) (Vila-Aiub et al. 2012) which is included in this study. Interestingly, this mechanism class has been also reported for *Sorghum halepense* in USA soybean (Riar et al. 2011). However, *Sorghum halepense* with glyphosate resistance evolved not only in Salta but also in other different areas in northern and central Argentina where transgenic glyphosate-resistant soybean is grown. It has first been reported in the provinces of Salta, Tucumán and Santiago del Estero. It has been rapidly extended to the south including the pampas (Vila-Aiub et al. 2007; Valverde and Gressel 2006; see Fig. 1).

**Figure 1:**
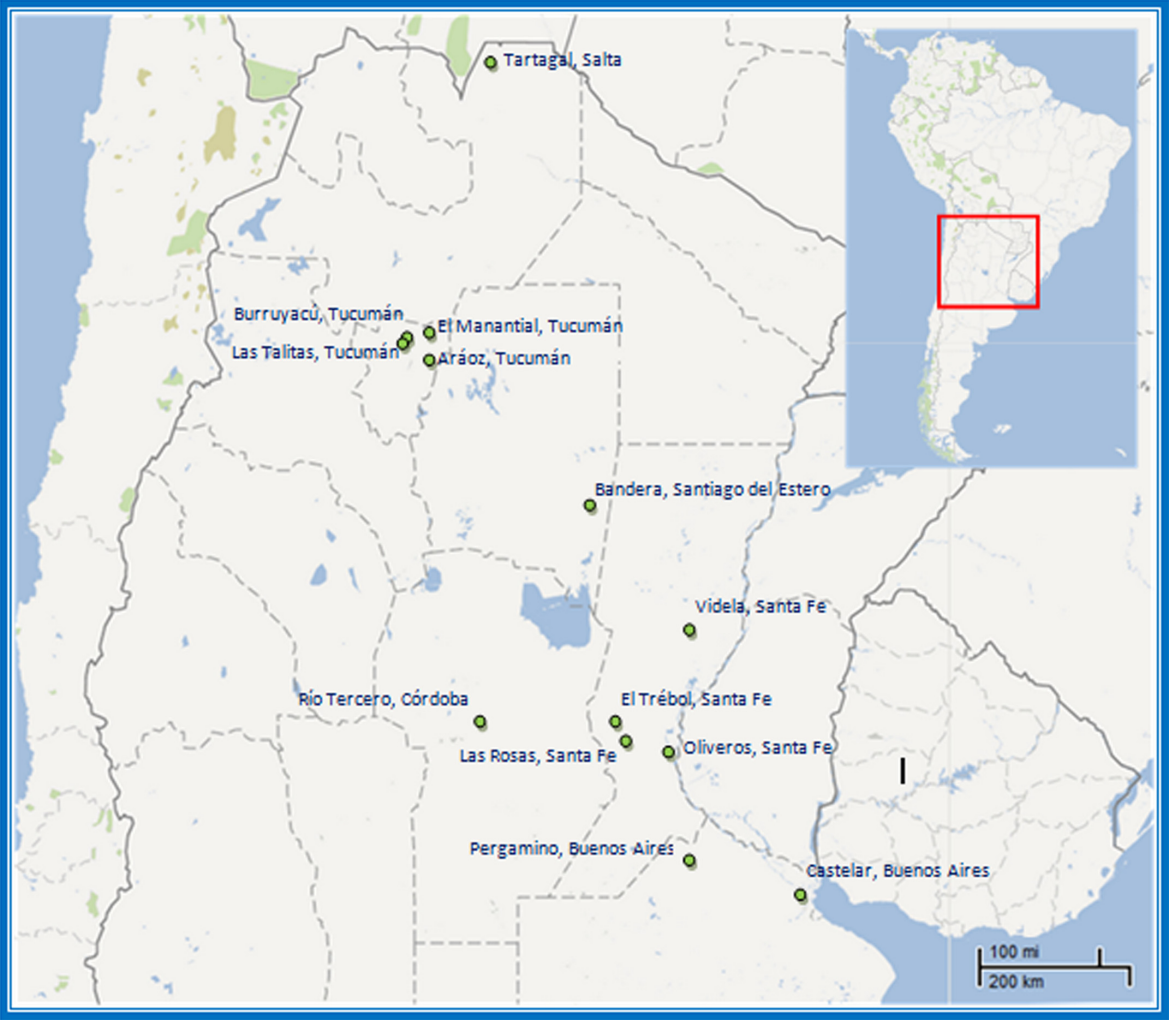
Geographic distribution of the collected Johnsongrass accessions. Map of Argentina showing collection sites of Johsongrass accessions used in this study and described in Table 1. The sites are indicated by circles. Dashes delimit the different provinces.

The aims of this study were (1) to adapt a set of genome-wide representative SSR markers with putative tetrasomic inheritance for population genetics analyses in *S. halepense* and study some important genetic parameters for this species; (2) more specifically, to analyze the genomic relationships between different glyphosate-resistant accessions and their susceptible neighboring counterparts; and (3) to verify if target-site resistance, as described for other glyphosate-resistant weeds, is present in any of the accessions.

## Materials and Methods

### Plant material

*Sorghum halepense* accessions from the reported outbreak spots were provided by the herbologists belonging to public institutions supervised by SENASA (which is the Argentine governmental authority that finally certified the resistance status of the collected weed material and elaborated the official epidemiological map from which derived the Fig. 1). Forty six accessions were analyzed (Table 1). The original location of the rhizomes was recorded through GPS and, when possible, planted in security greenhouses at INTA Castelar. Fresh germinated leaves from rhizomes or leaf samples directly collected from the field were used for DNA and RNA extraction.

**Table 1:** List and place of collection of Johsongrass samples

ID	Collection site	Latitude	Longitude
BAs01	Castelar, Buenos Aires	34°37'20.32″S	58°39'15.08″W
BAs02	Pergamino, Buenos Aires	33°56'40.64″S	60°36'54.44″W
COr01	Río Tercero, Córdoba	32°12'35.57″S	64°08'42.51″W
COs02	Río Tercero, Córdoba	32°13'5.91″S	64°08'52.01″W
SAr01	Tartagal, Salta	22°41'4.11″S	64°02'58.73″W
SAr02	Tartagal, Salta	22°49'1.15″S	64°02'59.54″W
SAr03	Tartagal, Salta	22°41'36.80″S	63°37'43.23″W
SAr04	Tartagal, Salta	22°55'21.83″S	63°38'36.40″W
SAr06	Tartagal, Salta	22°40'06.10''S	63°47'14.80''W
SAs05	Tartagal, Salta	23°20'6.11″S	64°13'14.19″W
SEr01	Taboada, Santiago del Estero	28° 3'53.33″S	63°46'31.48″W
SEr02	Taboada, Santiago del Estero	28° 3'36.41″S	63°47'40.89″W
SEr03	Taboada, Santiago del Estero	28° 5'11.02″S	63°46'42.42″W
SEr04	Taboada, Santiago del Estero	28° 5'3.50″S	63°48'11.80″W
SFr01	Las Rosas, Santa Fe	32°26'9.60″S	61°34'27.32″W
SFr02	Las Rosas, Santa Fe	32°25'33.24″S	61°35'3.93″W
SFr03	Las Rosas, Santa Fe	32°26'7.42″S	61°36'17.41″W
SFr09	Videla, Santa Fe	30°55'3.85″S	60°39'13.34″W
SFr10	Videla, Santa Fe	30°55'8.49″S	60°40'1.27″W
SFr11	Videla, Santa Fe	30°55'43.72″S	60°40'4.51″W
SFr12	Videla, Santa Fe	30°57'0.18″S	60°36'11.71″W
SFr13	El Trébol, Santa Fe	32°13'21.71″S	61°42'23.87″W
SFr14	El Trébol, Santa Fe	32°13'8.72″S	61°43'32.79″W
SFr15	El Trébol, Santa Fe	32°12'15.67″S	61°43'35.19″W
SFr16	El Trébol, Santa Fe	32°13'10.46″S	61°42'25.39″W
SFs04	Oliveros, Santa Fe	32°32'45.17″S	60°51'24.58″W
SFs05	Oliveros, Santa Fe	32°32'30.85″S	60°50'49.83″W
SFs06	Oliveros, Santa Fe	32°32'0.37″S	60°51'24.20″W
SFs07	Oliveros, Santa Fe	32°31'58.25″S	60°52'1.19″W
SFs08	Videla, Santa Fe	30°56'26.19″S	60°39'02.78″W
TUr01	Aráoz, Tucumán	27°02'43.40''S	64°53'55.90''W
TUr02	Aráoz, Tucumán	27°02'41.00''S	64°53'55.80''W
TUr03	Aráoz, Tucumán	27°02'2.36″S	64°54'35.24″W
TUr06	Aráoz, Tucumán	27°02'41.24''S	64°53'55.17''W
TUr07	Aráoz, Tucumán	27°01'52.65″S	64°55'17.54″W
TUr08	Aráoz, Tucumán	27°04'52.75''S	64°54'30.20''W
TUr09	Burruyacú, Tucumán	26°40'45.62''S	64°37'50.59''W
TUr10	Burruyacú, Tucumán	26°23'32.89''S	64°35'32.82''W
TUr11	Burruyacú, Tucumán	26°32'44.19''S	64°41'01.17''W
TUr15	Burruyacú, Tucumán	26°40'45.62''S	64°37'50.59''W
TUr17	Burruyacú, Tucumán	26°47'34.70''S	64°40'16.00''W
TUs04	EEA Obispo Colombres, Tucumán	26°46'24.52″S	65°12'02.86″W
TUs05	Las Talitas, Tucumán	26°47'18.20''S	65°12'21.00''W
TUs12	Burruyacú, Tucumán	26°19'52.90''S	64°36'55,20''W
TUs13	Las Talitas, Tucumán	26°47'18,20''S	65°12'21.00''W
TUs16	El Manantial, Tucumán	26°51'0.11″S	65°18'23.37″W

Sample ID codification: the first two capital letters denote the Province origin; third letter indicates resistance (r) or susceptibility (s) to glyphosate.

Two characterized *S. bicolor* inbred lines (IS9530 and RedLand) were used as outgroups and controls for molecular marker profiling.

### PCR amplification of SSR loci

Genomic DNA was extracted from fresh leaves by grinding with pestle and mortar under liquid nitrogen and extracted using Qiagen DNEasy Plant Mini Kit (Qiagen, Hilden, Germany) as suggested by the provider. DNA concentration was measured using a NanoDrop Spectrophotometer (NanoDrop Tech-Nologies, Wilmington, DE) following supplier instructions. Primers were synthesized by Alpha DNA (Montreal, Quebec, Canada). PCR conditions were as follows: a final volume of 12 µL containing 20 ng of genomic DNA, 0.25 µmol/L of each primer, 2 mmol/L MgCl_2_, 0.2 mmol/L of each dNTP, 1X reaction buffer, and 1 U Platinum Taq polymerase (Invitrogen, Grand Island, NY) using a Mastercycler ep-gradient thermocycler (Eppendorf, Hamburg, Germany). Amplifications were performed with the following touchdown program: denaturation step of 15 min at 94°C, followed by 10 cycles of 10 sec at 94°C, 20 s at 61°C reducing 1°C each cycle, and 30 sec at 72°C. This program was followed by a second phase of 31 cycles of 10 sec at 94°C, 20 sec at 54°C, and 30 sec at 72°C. The final extension step was of 20 min at 72°C. Amplified sequences ranged from 100 to 350 bp. A total of 30 different primer combinations were assayed and 12 were selected on the basis of their performance, PIC (Polymorphic information content) value, and number of amplified bands. Details of selected primer sequences and SSR location (SSR loci were mapped in different ligation groups of *S. bicolor* and used in gene/QTL mapping (Bhattramakki et al. 2000; Kong et al. 2000; Li et al. 2009; Mace et al. 2009; Srinivas et al. 2009; Mace and Jordan 2010; among others) are described in Table 2.

**Table 2:** Characterization of the selected single sequence repeats (SSR) markers

Name	Primer sequences (Forward/Reverse)	Chromosome	Alleles	Alleles/plant	PIC	Ho
*Xgap57*	ACAGGGCTTTAGGGAAATCG CCATCACCGTCGGCATCT	1	10	1.66	0.86	0.48
*Xtxp298*	GCATGTGTCAGATGATCTGGTGA GCTGTTAGCTTCTTCTAATCGTCGGT	2	20	2.65	0.91	0.93
*Xtxp4*	TGATGTTGTTACCCTTCT AGCCTATGTATGTGTTCGTCC	2	15	2.51	0.91	0.89
*Xtxp50*	TGATGTTGTTACCCTTCTGG AGCCTATGTATGTGTTCGTCC	2	12	2.46	0.80	0.89
*Xtxp63*	CCAACCGCGTCGCTGATG GTGGACTCTGTCGGGGCACTG	2	11	2.15	0.85	0.84
*Xtxp16*	TAGGGAAGAGCAAGTGCAGAC AAGAAAGGGCCCAGAGTTTC	3	13	2.31	0.82	0.89
*Xtxp27*	AACCTTGCCCTATCCACCTC TATGATGAATCAAGGGAGAGG	4	10	2.28	0.86	0.89
*Xtxp123*	TCGGCGAGCATCTTACA TACGTAGGCGGTTGGATT	5	5	1.87	0.60	0.70
*Xtxp303*	AATGAGGAAAATATGAAACAAGTACCAA AATAACAAGCGCAACTATATGAACAATAAA	5	9	2.70	0.80	0.96
*Xtxp295*	AAATCATGCATCCATGTTCGTCTTC CTCCCGCTACAAGAGTACATTCATAGCTTA	7	17	2.73	0.90	0.93
*Xtxp47*	CAATGGCTTGCACATGTCCTA GGTGCGAGCTAGTTAAGTGGG	8	14	1.77	0.79	0.62
*Xtxp67*	CAATGGCTTGCACATGTC GGTGCGAGCTAGTTAAGTGGG	9	16	2.76	0.90	0.91
Total/Average	–	–	152	2.32	0.83	0.83

First column catalogs heterologous public SSR markers developed for *S. bicolor*. The adjacent sequences used for PCR primer design are depicted in the second column. Third column indicates SSR loci location. Fourth and fifth columns show the maximum number of alleles and the average number of alleles per plant in the 48 different plants analyzed in this work, respectively. The last 2 columns show PIC and observed (Ho) heterozygosis, respectively. The last row indicates the total number of alleles and the average of the upper individual values.

Amplified polymorphic fragments from genomic SSR were evaluated by labeling the forward primer with a 6-FAM, HEX, or NED fluorescent dye (Applied Biosystems, Foster City, CA and Alpha DNA) and separated on an ABI3100 Genetic Analyzer (Applied Biosystems). Fragment sizing was done using the ROX 500 internal-lane standard (ROX, 6-carboxy-X-rhodamine; Applied Biosystems). GeneMapper® Software Version 4.0 (Applied Biosystems) was used to score SSR alleles. Allele assignments were made by size comparison with the standard allelic ladders, using the ID software provided by Applied Biosystems.

### Genetic analyses

Microsatellite marker scoring presents difficulties in polyploid species because it is usually very difficult to assess which allele(s) occur in more than one copy or the true allelic relationship (homologous and homeologous) at each of the microsatellite loci. For these reasons, the microsatellites were treated as dominant markers. Accordingly, data were scored based on the presence (1) or absence (0) of a band (Andreakis et al. 2009; Arroyo et al. 2010; Vigna et al. 2011). Genotypic data (bands) were converted to dominant loci as described in Rodzen et al. (2004). Each individual's genotype is converted into a 1× *n* vector where n is the number of bands at the locus. For each band *i* in the system, the *i*th element in the vector is assigned a value of 1 if that band is present or a value of 0 if that band is absent in that sample's genotype. (For example, a microsatellite with a six-allele genotype is converted to a 1 × *n* vector of [0 1 1 0 1 0], yielding six dominant markers). For more than one microsatellite locus, the process is repeated for each locus *j*. This produces a 1 × *nj* matrix for each locus, where *nj* is the number of bands at locus *j*. These matrices are then horizontally concatenated, resulting in a single 1 × *nT* matrix where *nT* is the total number of bands across all loci. Finally, data for all *k* individuals in the data set are concatenated in a *k* × *nT* matrix. This matrix now contains dominant marker data for all individuals and all microsatellite loci.

The binary data matrix was converted to a pairwise similarity matrix using the Jaccard association index that was subjected to cluster analysis using the UPGMA (unweighted pair-group method with arithmetic average) algorithm on NTSYSpc, version 2.10e (Rohlf 2000). The association between genetic differentiation and geographic distance was determined using Mantel tests (Mantel 1967) implemented in the software mentioned above.

Because of the tetraploid nature of *S. halepense*, allele frequencies and heterozygosis could not be computed in the conventional way done in diploid species using the canonic heterozygosis formula (Nei 1973). Instead, like in other polyploid species such as potato or sweet potato, heterozygosis was calculated by using the percentage of multiallelic accessions (Bonierbale et al. 1993; Zhang et al. 2000). PIC was defined as PIC = 1 − Σ *P*_*i*_^2^ where *P*_*i*_ is the frequency of the ith allele in the pool of total alleles for that locus.

Genetic relationships were also examined applying the Discriminant Analysis of Principal Components (DAPC), a multivariate method designed to identify and describe clusters of genetically related individuals, which is free of assumptions regarding Hardy–Weinberg or linkage equilibrium. Briefly, the method relies on allele data transformation using Principal Component Analysis (PCA) as a prior step to Discriminant Analysis (DA). DA defines a model in which genetic variation is partitioned into a between-group and a within-group component. Groups can be defined a priori (i.e., populations, collection sites, temporal affiliations, etc.) or can be inferred using first sequential K-means (Legendre and Legendre 1998) and model selection. This analysis was performed using the Adegenet package (Jombart 2008) for the R 2.10.1 software (R development Core Team, 2009). The function “DAPC” was executed using the clusters identified by K-means. The number of clusters was assessed using the function “find.clusters,” evaluating a range from 1 to 40. The optimal number of clusters was chosen on the basis of the lowest associated Bayesian Information Criterion (BIC). Fifteen principal components were retained for data transformation.

### Nucleotide sequencing of EPSPS encoding genes

Total RNA or DNA was isolated from leaf tissue of individual plants obtained from field-collected rhizomes as described in Vila-Aiub et al. (2012) or in Lijavetzky et al. (2000), respectively. RNA quality was assessed using the Agilent 2100 Bioanalyzer (Agilent Technologies, Santa Clara, CA) following the manufacturer's guidelines. Genomic DNA concentration was estimated as described above using a Nanodrop spectrophotometer.

The *EPSPS* coding sequence of BAS01 was amplified by RT-PCR from cDNA and sequenced as described in Vila Aiub et al. (2012).

PCR reactions from genomic DNA were performed with primers: EPSPS1-F 5' TGAGGATGTTCACTACATGC 3' and EPSPS1-R 5' GTGCTGCAATTACTGGAGG 3'. Amplifications were carried out in 25 μL of 1 × PCR buffer, .0.2 µmol/L of each primer, 1.5 mmol/L MgCl_2_, 0.2 mmol/L of each dNTP, and 1.5 U Platinum *Taq* polymerase (Invitrogen) using a Mastercycler ep-gradient thermocycler (Eppendorf). Amplifications were performed following this program: denaturation step of 4 min at 94°C, followed by 31 cycles of 45 sec at 94°C, 1 min at 54°C, and 1 min at 72°C. The final extension step was of 10 min at 72°C. Amplified products derived from genomic DNA were sequenced without cloning using an ABI 3730 XL automated sequencer. Nucleotide sequences were assembled and compared among resistant and susceptible plants using Vector NTI Suite 8.0 software (InforMax Inc., Bethesda, MD). Twenty one of the 40 nucleotide sequences used in this study were at GenBank under accession numbers: HQ436351 (SAr02), HQ436352 (SAr03), HQ436353 (SAs05) and HQ436354 (BAs02), KC222054 (SEr01), KC222055 (TUr15), KC222056 (TUr03), KC222057 (SFs07), KC222058 (COs02), KC222059 (TUr02), KC222060 (TUs13), KC222061 (TUr01), KC222062 (SFs05), KC222063 (SFr10), KC222064 (SFs08), KC222065 (TUr11), KC222066 (SEr04), KC222067 (TUs16), KC222068 (SFs06), KC222069 (SFr12), and KC914621 (BAs01).

The rest of the sequences were not deposited because they fail to fit the requirements of GenBank (length and number of “N” allowed).

## Results and Discussion

### Transferability of heterologous *S. bicolor* SSR markers to *S. halepense*

DNA simple sequence repeats (SSRs) are robust and very informative codominant genetic markers that can be easily amplified with PCR and detected by capillary gel electrophoresis in an automated nucleotide sequencer. For *S. bicolor*, as many as 7000 putative SSR markers were reported and contributed to the construction of many different saturated genetic maps, like the ones recently reported by Li et al. (2009), Mace et al. (2009), or Ramu et al. (2010), some of which were readily adapted to Johnsongrass (Chang et al. 2007; Wu and Huang 2006; Guo et al. 2008; Hopp et al. 2010; Jessup et al. 2012). This is because *S. halepense* is an allotetraploid containing one subgenome which is homologous to *S. bicolor* and another subgenome that originated from *S. propinquum*. In addition, it was reported that active gene flow from cultivated sorghum to Johnsongrass is occurring (Morrell et al. 2005). Accordingly, heterologous SSR transference from *S. bicolor* to *S. halepense* was successful for all the SSR loci that were assayed here. However, some SSR primers amplified up to two alleles per plant, whereas others amplified up to four alleles per plant. In addition, some of the different assayed plants depicted only one SSR band (of the four possible ones), suggesting the occurrence of null alleles. Although mutations affecting the comparatively more conserved sequences adjacent to SSR may occur in any of the two subgenomes, owing the evolutionary divergence between *S. bicolor* and *S. propinquum*, null alleles are probably principally located in *S. propinquum* genome, since the SSR were specifically developed for *S. bicolor*. In order to have a wider coverage of Johnsongrass genome, among the 30 different SSR loci assayed for this work, only those putatively able to evidence alleles in both homeologous genomes were selected for the following described genetic studies. All selected SSR markers were highly polymorphic, some of them evidenced up to 20 different alleles in the samples studied (see Table 2).

### Selected markers reveal unexpected polymorphism levels

Twelve SSR markers reporting information of 24 genome-wide distributed loci that generated up to four bands per plant were selected to evaluate the genetic diversity of 46 geographically distributed Johnsongrass accessions. Two very different cultivated sorghum inbred lines were included as external controls in the comparisons. Polymorphic allelic patterns and PIC values per primer pair and their comparison with the parameters reported for *S. bicolor* are represented in Table 2. Selected SSR primer pairs yielded 152 alleles with a range of 5–20 alleles per marker. SSR loci *Xtxp4* and *Xtxp298* revealed the highest PIC value (0.90), whereas *Xtxp123* had the lowest (0.60) resulting in an average PIC value of 0.83. According to Vaiman et al. (1994), loci polymorphism can be considered high, medium, or low if PIC > 0.5, PIC > 0.25, and PIC < 0.25, respectively. In Johnsongrass genotypes, all the selected SSR markers revealed a PIC > 0.5. These results indicate that these markers are highly informative and very useful to study the genetic structure of Johnsongrass.

The observed heterozygosis levels varied across the 12 loci, ranging from 0.48 to 0.93, with a mean of 0.83. The observed heterozygosity was reported to range from 0.477 to 0.502 in the tetraploid *Solanum tuberosum* (Bonierbale et al.^1993^) and from 0.31 to 0.92 with a mean of 0.60 in hexaploid sweetpotato (Zhang et al. 2000). In Johngrass, it was reported that less than 5% of fertilized plants are the result of outcrossing, even in fields where plants are closely spaced (Tarr 1962).

However, these results were obtained at a time when molecular tools for estimating genetic diversity (like SSR) were not available. Therefore, the results reported here strongly suggest that cross-pollination is much more important in Johsongrass reproductive strategy than thought before.

### Similarity analysis reveals geographic clustering

**Figure 2:**
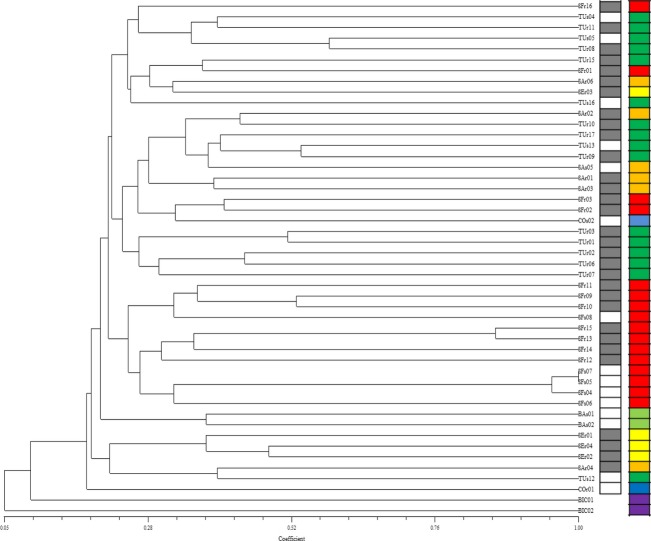
UPGMA dendrogram based on Jaccard's similarity coefficient from 12 SSR marker data showing genetic relationships among the 46 *Sorghum halepense* samples. Accessions are codified as indicated in Table 1. External controls used are diploid *S. bicolor* imbred lines IS9530 and Red Land. Rectangles gray and white: samples resistant and suceptible to glyphosate, respectively. Rectangles in colors represent the geographic origin: 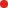
Santa Fé, 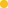
Salta, 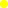
Santiago del Estero, 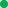
Tucumán, 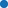
Córdoba, 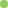
Buenos Aires, 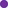
outgroup: *Sorgum bicolor*.

A total of 152 scorable alleles were obtained. A dendrogram was constructed using UPGMA, based on the Jaccard genetic similarity (GS) coefficient. The resulting tree plot is shown in Figure 2. The dendrogram shows distinct genetic relationships between Johnsongrass accessions. The greatest genetic distance was found between the two *S. bicolor* external controls and the remaining OTUs (GS = 0.05–0.10), which indicates that the genetic similarity is relatively low and supports the choice of *S. bicolor* as a suitable external control in the phenogram. This result was not completely expected, since *S. halepense* shares one subgenome with *S. bicolor* and because it was reported that genetic flow between the two species actively occurs through hybridization followed by introgression (Morrell et al. 2005). This happens not only in Johnsongrass populations adjacent to long-term sorghum production fields in Texas and Nebraska but also, to a lesser extent, in Johnsongrass populations from New Jersey and Georgia with no recent exposure to cultivated sorghum (Morrell et al. 2005).

Since the molecular markers used in our work (SSR) have a much faster evolutionary rate than the rather more conserved ones (i.e., RFLP) used by Morrell et al. (2005), it is difficult to conclude whether such a recent introgression occurred in Argentina or not.

The smallest genetic distances were observed between two almost identical neighboring plants (from Oliveros, Province of Santa Fe), although most of the accessions showed comparatively low similarity values (<0.5) reflecting a rather important genetic diversity. Jaccard similarity and cophenetic matrices showed a correlation of 0.791 suggesting that the tree plot adequately represents the similarity matrix without any major distortion. The 46 accessions were clustered into, at least, seven geographically groups (with GS larger than 0.3). While most provenances showed to group into one major cluster, each of the accessions from the Province of Tucumán were separated into three groups. Since clustering according to geographic origin was evident, but not fully conclusive, a new matrix including geographic distances was constructed. Using Mantel (1963) algorithm, a low but positive correlation (*r* = 0.22514, *P* < 0.002) confirmed the relationships suggested by the dendrogram.

**Figure 3:**
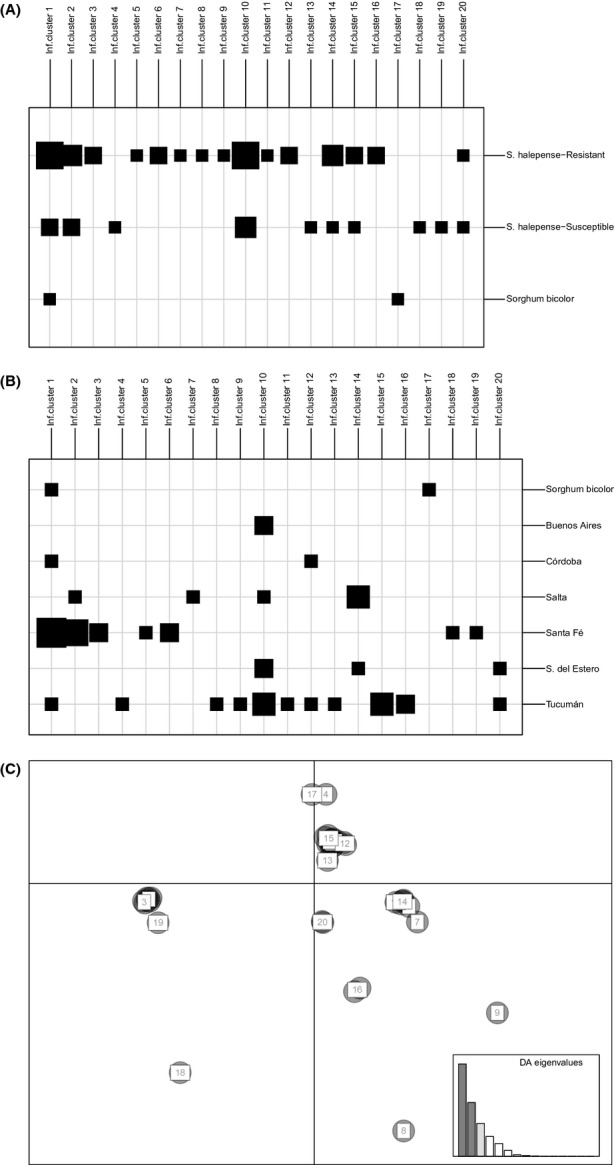
Discriminant Analysis of Principal Components (DAPC) of *Sorghum halepense* samples. Scatterplot of the DAPC identified by the K-means algorithm using the 20 clusters with regard to glyphosate resistance/susceptibility (A) and province of origin (B). Insets indicate the number of principal components retained for DAPC. (C) Cluster assignment according to the K-means algorithm. The represented sizes of the squares are proportional to the number of individuals in each group. Sample assignment to the 20 clusters used for DAPC analysis grouping the *S. halepense* samples as follows: Cluster 1: SFr11, BIC01, COs02, TUr02, SFr14, SFr12, SFr10, SFs08; Cluster 2: SFr16, SAr01, SFr01, SFs06, SFs04; Cluster 3: SFr03, SFr02; Cluster 4: TUs04; Cluster 5: SFr09; Cluster 6: SFr13, SFr15; Cluster 7: SAr03; Cluster 8:TUr06; Cluster 9: TUr09; Cluster 10: SEr04, BAs01, TUr17, SAr06, TUs12, TUr10, BAs02, SEr02; Cluster 11: TUr15; Cluster 12: COr01, TUr01; Cluster 13: TUs16; Cluster 14: SAr02, SEr01, SAr04, SAs05; Cluster 15: TUs05, TUr08, TUr11; Cluster 16: TUr03, TUr07; Cluster 17: BIC02; Cluster 18: SFs05; Cluster 19: SFs07; Cluster 20: TUs13, SEr03.

Interestingly, the 32 glyphosate-resistant accessions did not cluster together. In contrast, they occurred dispersed along the phenogram. Similarity values between glyphosate-resistant accessions varied from 0.52 to 0.1 (which is the largest intraspecific distance within the matrix). Genetic relationships were also examined applying the DAPC (Jombart et al. 2010). The DAPC scatterplot using the clusters identified by K-means did not show a clear pattern of association among samples by considering either resistance status or province of origin (Fig. 3A). Indeed, clusters 1, 2, 10, 14, 15, and 20 were composed of both resistant and susceptible individuals (Fig. 3B) and a similar configuration was found when analyzing cluster assignment among provinces, although a subtle geographic structuring seems to be apparent (Fig. 3C).

Some ecological features of Johnsongrass like seed dispersion capacity by birds as well as agricultural practices, leading to agricultural crop seed contamination with Johnsongrass seeds would tend to predict a fluid genetic flow in rather extended areas in detriment of geographic subpopulations. These factors would promote a rapid dispersion and establishment of a strong adaptive trait like herbicide resistance, along with a genetic drift owing to the strong negative selection that occurred with susceptible genotypes. This population dynamic model, together with a very low probability of occurrence of a mutation conferring glyphosate resistance, supports a single origin hypothesis of resistance genes. However, as shown above, clustering analysis based on the genetic backgrounds of the sampled plants showed that they are structured in geographic distances. Furthermore, glyphosate-resistant genotypes do not share similar genetic backgrounds, but, on the contrary, show closer similarity to their neighboring susceptible genotypes than to other resistant accessions. These results do not support a single genetic origin of glyphosate resistance.

Although the pattern along the country evidences the absence of a single origin, this kind of dispersion of the resistance may be occurring at the local level. The samples TUr09, TUr10 and TUr17 that come from different farms are in the same cluster and, in this case, it is known that these samples may be related by the same harvesters.

### Nucleotide sequencing of EPSPS encoding gene shows no evidence of expected target-site resistance mechanisms

As recently reported by our group, the full *S. halepense* EPSPS coding sequence comprises 1335 kb, encoding 444 amino acids (Vila-Aiub et al. 2012). Encoded aminoacid sequences have 99% identity with *S. bicolor* EPSPS. Partial EPSPS genomic DNA sequencing of 36 different *S. halepense* accessions revealed polymorphisms in both nucleotides and deduced amino acid sequences. Nucleotide sequencing also revealed other strong differences like an INDEL of three nucleotides which is responsible for an extra aminoacid insertion in EPSPS sequence in a susceptible accession collected at Castelar (Province of Buenos Aires). However, in none of the 32 resistant accessions, there were amino acid changes in the known resistance mutation sites from codons 101 to 106 (Fig. S1). Furthermore, of the other nucleotide polymorphisms detected within the EPSPS coding and noncoding sequences (intron sequences), none of them showed association with glyphosate resistance, suggesting that glyphosate resistance determinant is probably not located at or nearby the EPSPS locus. Genetic analysis of a large number of segregating populations derived from selfing glyphosate-resistant *S. halepense* is in progress to confirm this hypothesis.

Therefore, the mutations in the EPSPS gene known to confer glyphosate resistance in some annual weeds are not present in the assessed *S. halepense* genotypes. Recently published results show that *S. halepense* plants from Salta (accessions SAr01-04) exhibit a nontarget mechanism of resistance consisting in reduced rates of glyphosate translocation from leaf to crown and root tissues (Vila-Aiub et al. 2012). The results of this work reveal that glyphosate resistance in the 32 accessions of Argentinean *S. halepense* evaluated here are likewise endowed by nontarget-site resistance mechanisms. However, it is not possible to ensure whether or not the mechanism in all sequenced accessions is the same to the one described by Vila-Aiub et al. (2012).

In brief, this work demonstrates the feasibility of adapting sorghum microsatellite markers for studying the genetic structure of Johnsongrass populations, some of which are strictly located in its homologous subgenome and others are able to detect the homeologous loci coming from *S. propinquum*. It also shows that outcrossing is important in Johnsongrass reproductive strategy and samples are structured according to their geographic distances. Moreover, we conclude that glyphosate-resistant genotypes do not share similar genetic backgrounds between them, but, on the contrary, they show similarity to their neighboring susceptible counterparts. In agreement with the previous conclusion, genetic structure revealed by DAPC did not show a clear pattern of association among samples considering either resistance status or province of origin. Sequencing of the EPSPS-encoding gene from glyphosate-resistant genotypes collected from different geographic origins was carried out. This not only confirmed our previous report in which characterized accessions from Salta do not present mutations in aminoacid positions 101 or 106 (Vila-Aiub et al. 2012), but, interestingly, it also showed that none of the other geographic isolates presented these otherwise expected mutations.

## Supporting Information

**Figure S1.** Nucleotide sequences of EPSPS isolated from glyphosate-resistant and glyphosate-susceptible *S. halepense* genotypes. Multiple alignments of nucleotides located in the most significant segment of the *EPSPS* gene belonging to 36 different sample sequences including the critical 101 and 106 codons (marked positions).

**Table S1.** SSR genotypes. Matrices containing SSR data: the 0 1 matrix and the matrix with the allele's molecular weight.
