# Supplementary figures and images for: Population genetics structure of glyphosate-resistant Johnsongrass (Sorghum halepense L. Pers) does not support a single origin of the resistance
Source: Ecol Evol. 2013 Aug 24;3(10):3388–400. doi: 10.1002/ece3.671 (PMC3797486; doi:10.1002/ece3.671)

Figure S1

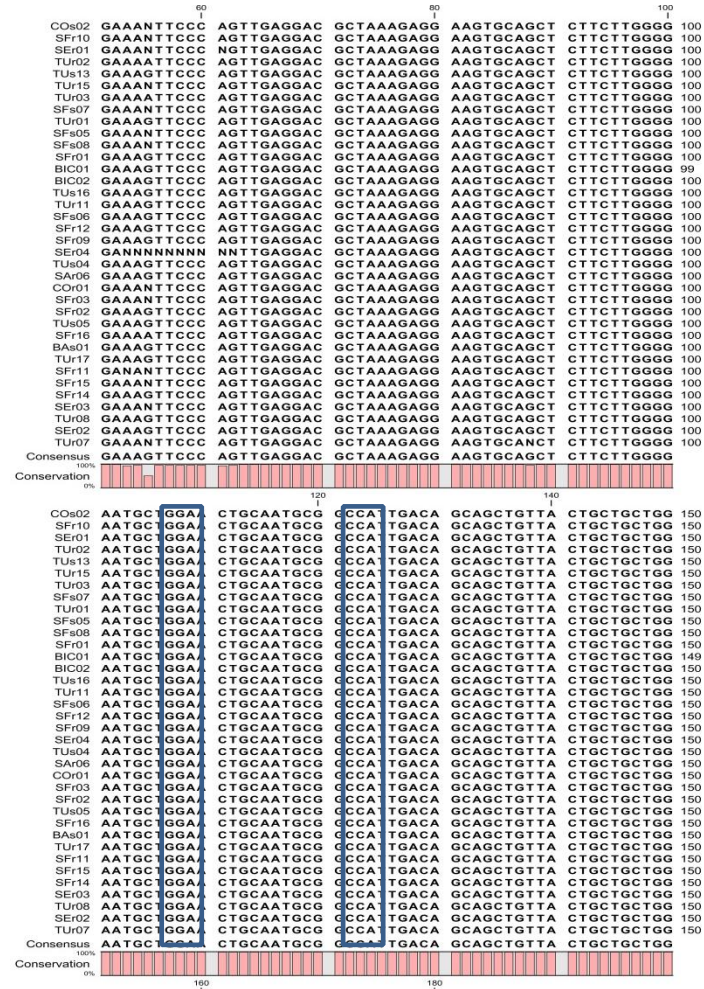

Supplement: Supplementary file 1 [file ece30003-3388-SD1.pdf]
